# *SNORD116* and growth hormone therapy impact IGFBP7 in Prader–Willi syndrome

**DOI:** 10.1038/s41436-021-01185-y

**Published:** 2021-05-26

**Authors:** Sanaa Eddiry, Gwenaelle Diene, Catherine Molinas, Juliette Salles, Françoise Conte Auriol, Isabelle Gennero, Eric Bieth, Boris V. Skryabin, Timofey S. Rozhdestvensky, Lisa C. Burnett, Rudolph L. Leibel, Maithé Tauber, Jean Pierre Salles

**Affiliations:** 1 Centre de Physiopathologie de Toulouse Purpan, CPTP, UMR INSERM 1043 CNRS 5828, Université Paul Sabatier, Toulouse, France; 2 Unité de Recherche Clinique Pédiatrique CIC 1436, Hôpital des Enfants, CHU de Toulouse, Toulouse, France; 3 Centre de Référence du Syndrome de Prader–Willi, Toulouse, France; 4 Unité d’Endocrinologie, Hôpital des Enfants, CHU de Toulouse, Toulouse, France; 5 Service de Psychiatrie et Psychologie, CHU de Toulouse, Toulouse, France; 6 Génétique Médicale, CHU Toulouse-Purpan, Hôpital Purpan, Toulouse, France; 7 Medical Faculty, Core Facility Transgenic Animal and Genetic Engineering Models (TRAM), University of Muenster, Muenster, Germany; 8 Levo Therapeutics, Skokie, IL USA; 9 Department of Pediatrics, Division of Molecular Genetics, Columbia University, NY New York, USA

## Abstract

**Purpose:**

Prader–Willi syndrome (PWS) is a neurodevelopmental disorder with hypothalamic dysfunction due to deficiency of imprinted genes located on the 15q11-q13 chromosome. Among them, the *SNORD116* gene appears critical for the expression of the PWS phenotype. We aimed to clarify the role of *SNORD116* in cellular and animal models with regard to growth hormone therapy (GHT), the main approved treatment for PWS.

**Methods:**

We collected serum and induced pluripotent stem cells (iPSCs) from GH-treated PWS patients to differentiate into dopaminergic neurons, and in parallel used a *Snord116* knockout mouse model. We analyzed the expression of factors potentially linked to GH responsiveness.

**Results:**

We found elevated levels of circulating IGFBP7 in naive PWS patients, with IGFBP7 levels normalizing under GHT. We found elevated IGFBP7 levels in the brains of *Snord116* knockout mice and in iPSC-derived neurons from a SNORD116-deleted PWS patient. High circulating levels of IGFBP7 in PWS patients may result from both increased *IGFBP7* expression and decreased IGFBP7 cleavage, by downregulation of the proconvertase PC1.

**Conclusion:**

*SNORD116* deletion affects IGFBP7 levels, while IGFBP7 decreases under GHT in PWS patients. Modulation of the IGFBP7 level, which interacts with IGF1, has implications in the pathophysiology and management of PWS under GHT.

**Graphical Abstract:**

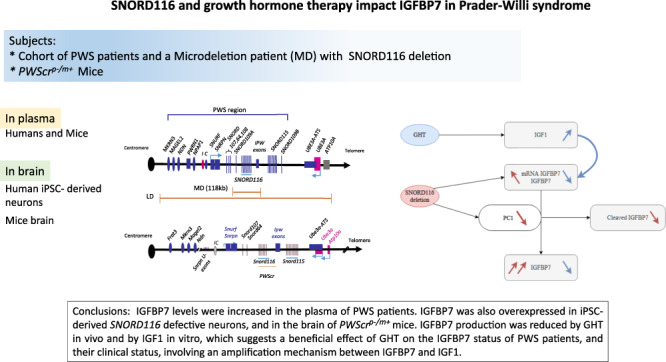

## INTRODUCTION

Prader–Willi syndrome (PWS) is a neurodevelopmental disorder (approximately 1 to 20,000 live births), due to deletions of the paternal chromosomal region 15q11-q13, maternal disomy, or an imprinting defect.^1^ Early hallmarks are motor delay, poor sucking and swallowing, and somnolence with anorexia. Later in childhood appear learning disabilities and disruptive behavioral problems, with hyperphagia leading to severe obesity.

Reports have described patients with a PWS phenotype associated with microdeletions including small nucleolar C/D box RNAs (*SNORD*).^2–6^ This suggested a minimal critical region (MCR) including *SNORD116* region sufficient to cause PWS phenotype. The *SNORD116* region generates non-protein-coding exons as the *SNORD116* host gene (116HG) and non-protein-coding transcripts as intronically embedded the *SNORD116* small nucleolar RNAs.^7^

We have reported a case of a typical PWS phenotype with microdeletion (MD) (118 kb; 15q:15:25,257,217–15:25,375,376) removing the *SNORD109A*, the *SNORD116* cluster, and the *IPW* gene,^5^ which corresponds to the MCR. Induced pluripotent stem cell (iPSC)-derived neurons from this patient enabled the development of a hypothesis regarding the origin of neuroendocrine defects of PWS patients. A decrease in *NHLH2* (Nescient Helix-Loop-Helix 2) and *PCSK1* (ProConvertase Subtilisin/Kexin type 1) expression in the iPSC-derived neurons resulted in altered prohormone processing.^8^ Hypothalamic loss of the *Snord116* cluster in mice was also shown to reproduce the hyperphagia phenotype observed in PWS, providing the first adult mouse model of PWS.^9^ Analysis of the hypothalamic transcriptome of PWS patients suggested that putative *SNORD116*-regulated genes influence neuronal loss.^10^

Growth hormone (GH) deficiency (GHD) is prominent in neuroendocrine dysfunctions of PWS and early GH therapy (GHT) is recommended.^1,11,12^ GHT hypersensitivity is likely in PWS, with a marked increase in insulin growth factor 1 (IGF1).^13^ GH binding proteins (GHBP) are potential regulators of the effect of GH and IGF1 under GHT. It is well-known that the IGF1, IGFBP3 and acid labile subunit (ALS) ternary complex modulates the level of the free active form of IGF1, thereby influencing the effect of IGF1 on the IGF1 receptor (IGF1R).^14^ IGFBP7, an IGFBP-related protein first described in the context of GH and insulin sensitivity^15,16^ also regulates the bioavailability of IGF1. IGFBP7 may block IGF1R activity by competing with IGF1.^17^ IGFBP7 is mainly regulated by its messenger RNA (mRNA) expression and by proteolysis involving an editing process, with intact and cleaved forms of IGFBP7 displaying different biological activities.^18–21^ In cancer, the level of expression of *IGFBP7* is variable depending on the cell type. IGFBP7 usually acts as suppressor gene,^22–24^ and less frequently as an oncogene.^14^ Finally, IGFBP7 is now considered to be involved in neuronal development and degeneration.^25,26^

Modulated levels of IGFBP7, which interacts with IGF1 and IGF1R, has therefore potential implications in the pathophysiology and management of PWS under GHT. Moreover, we formulated the hypothesis that *SNORD116* could be a regulator of *IGFBP7* expression in PWS.

Thus, we explored the IGFBP7 level as a potential factor of GHT sensitivity in GH-treated PWS patients. To better understand its potential role in neuronal degeneration we assessed iPSC-induced neurons with a deletion of the nonmethylated *SNORD116* allele (*SNORD116*-deleted) and a *Snord116* knockout murine model.

## MATERIALS AND METHODS

### Clinical measures and sample collections

Patient**s** and healthy control subjects were recruited from a clinical research program (clinical trial NCT01298180; n°EudraCT 2008-004612-12) approved by our institutional review board (the CPP Toulouse 1 Ethics Committee). Peripheral blood samples were from patients under GHT before and after one year of GHT starting at a dose of 0.035 mg/kg/day.

PWS diagnosis was established using the standard DNA methylation test. Patients as well as parents or carers gave their written consent before taking part in the study. Major exclusion criteria for GHT were:

- Children with a contraindication for growth hormone.
- Children with hypersensitivity to the active substance or any of the excipients of Genotropin^®^.

See Supplementary Methods for more details.

### Fibroblast reprogramming to iPSCs and differentiation into neurons

Primary human fibroblasts from unaffected controls and PWS patients were reprogrammed to iPSC using retrovirus reprogramming, Sendai virus reprogramming, or mRNA reprogramming. R.L.L.’s team provided iPSCs. The method for inducing the differentiation of iPSCs into neurons has been described previously.^27^ In brief, iPSC cultures were routinely retained on mouse embryonic fibroblasts (MEFs) (Globalstem) and cultured in human embryonic stem cell (ES) media. IPSCs were differentiated into neurons using a modified SMAD inhibition protocol from fibroblasts from PWS patients and control patients.^28^ Neurons were harvested on day 34 for RNA extraction. *TH, GIRK2*, *DRD2*, and *NR4A2* were tested to validate the neural differentiation (Supplementary Fig. S1). Primers used are detailed in Supplementary data (Supplementary Table S3). Cells were dissociated, stained with a CD56 antibody, and sorted using a BD Biosciences Cell Sorter. The detailed procedure is described in Supplementary Methods.

### IGF1, IGFBP3, and IGFBP7 biochemical assays

Blood sampling was taken after an overnight fast. All measurements were performed in the same laboratory. Analyses were performed in duplicate in the same assay. Plasma IGF1 levels were determined by a chemiluminescent immunoassay from IDS-ISIS. The intra- and interassay coefficient of variation (CVs) were 2.9% and 7.2%, respectively. Standards and controls were calibrated against the World Health Organization reference reagent for IGF1 code 87/518. Plasma IGFBP3 was evaluated using an IGFBP3 enzyme-linked immunosorbent assay (ELISA) kit (ab211652) (Abcam®, Cambridge, UK), a sandwich ELISA. Assay sensitivity was of 86.6 pg/ml, intra-assay coefficient was <3.1% and interassay CV was <6.5%. The values are expressed in standard deviation scores (SDS). SDS calculation was based on the Nichols Institute Diagnostics reference method by Brabant et al.^29^ Plasma IGFBP7 levels were determined with a commercial ELISA double-antibody sandwich kit against intact IGFBP7: SEB673Hu for human assay and SEB673Mu for murine assay (USCN Life Science Inc®, Houston, TX, now available from Wuhan USCN Business Wuhan, China). Assay sensitivity was of 3.2 ng/ml for IGFBP7 Hu and 28.7 pg/ml for IGFBP7 Mu, intra-assay coefficient of variation (CV) was <10%, and interassay CV was <12% for both assays. The immunogen of the antibodies in SEB673Hu and SEB673Mu is a recombinant IGFBP7 (Ser28-Thr264), which almost corresponds to the full length IGFBP7. Plasma samples were diluted tenfold for humans and 100-fold for mice. Pearson product-moment correlate ion coefficients (*r*) were used to assess the relationship between variables for PWS subjects.

### DXA analysis

Dual-energy X-ray absorptiometry (DXA) (Lunar iDXA–GE Healthcare, Boston, MA, USA) was performed following the manufacturer’s instructions to determine body composition (lean and fat mass) before and after one year of GHT. More information in Supplementary data.

### qRT-PCR and ddPCR in human cells

Total RNA was extracted from 10^6^ iPSC-derived cells using TRIzol® reagent (Invitrogen), according to the manufacturer’s instructions. The quality and integrity of the RNA obtained were assessed using an Agilent 2100 Bioanalyzer (Agilent Technologies) after a denaturing step at 70 °C for 2 minutes. RNA was reverse-transcribed into complementary DNA (cDNA) with the Super Array RT2 First Strand kit (SABiosciences, Qiagen) starting from 1 µg total RNA according to the manufacturer’s instructions. Amplification of the cDNA and detection of the target polymerase chain reaction (PCR) product were conducted in a Light Cycler 480 detection system (LC480, Roche Applied Science) using an RT2 profiler PCR array (SABiosciences, Qiagen) according to the manufacturer’s instructions. The quantitative reverse transcription PCR (qRT-PCR) results were analyzed with sequence detection software from Light Cycler 480 (Roche Applied Science). All primers used for qRT-PCR assays were validated with a 5-point standard curve. *TBP* (TATA-binding protein) was used as a housekeeping gene. The data shown represent the mean ΔCt with the respective standard deviation when ΔCt = Ct gene of interest – Ct *TBP*. The data represent results from three independent experiments. See Supplementary Methods for details on droplet digital PCR (ddPCR) reactions. Primers used are described in Table S3.

### Western blot analyses

The IGFBP7 antibody from Cell Signaling (97884) was directed against C-terminal IGFBP7, detecting total IGFBP7. Monoclonal antirabbit horseradish peroxidase (HRP)–conjugated antibodies and horseradish peroxidase–conjugated secondary antibodies were from Sigma-Aldrich Co. Blots were revealed by an enhanced chemiluminescence detection system (Amersham). All PWS cases and controls (CON) were analyzed in the same experiment. See Supplementary Methods for more details.

### Animals

Previous reports show the generation and genotyping of *PWScr*^*p−/m+*^ mice that harbor the Snord116 locus deletion.^30^ *PWScr*^*p−/m+*^ and wild-type (WT) mice were provided by B.V.S. and T.S.R., and were used for experiments on organs and plasma. Phenotypical abnormalities relative to the deletion are evident when the deleted allele is inherited paternally *PWScr*^*p−/m+*^. Organs and plasma from seven *PWScr*^*p−/m+*^ mice (three females and four males) and their WT littermates (five males and two females) were analyzed on postnatal day 7 (P7). All the procedures concerning the mice were performed in compliance with the guidelines for the welfare of experimental animals issued by the Federal Government of Germany and approved by the State Agency for Nature, Environment and Consumer Protection, North Rhine-Westphalia (Landesamt für Natur, Umwelt und Verbraucherschutz Nordrhein-Westfalen). Animals were kept in specific pathogen-free animal facilities. All breeding conditions and the weaning of pups were carried out as previously described.^30^

### qRT-PCR of mouse organs

Organs from P7 ad libitum fed mice were homogenized in QIAzol, followed by phenol chloroform separation and precipitation in 70% ethanol of the aqueous fraction, which was subsequently applied to QIAGEN RNeasy columns. DNase treatment was performed. qRT-PCR was performed as described for iPSC-derived neurons. For gene quantification, several endogenous controls were tested: *cdkn1a*, *gapdh*, *rpl27*, and *pgk1* but only *pgk1* was selected as it showed expression levels that remained relatively constant with low variance and high abundance across the samples that were tested. The data represent results of the RNA analysis from three independent experiments, 7 WT vs. 7 *PWScr*^*p−/m+*^ mice. The data shown represent the mean ΔCt with respective standard deviation when ΔCt = Ct gene of interest – Ct of *pgk1*.

### Statistics

Considering the sample size and the normality or absence of normality of the distribution of the different values, we used appropriate statistical tools. Comparisons between two groups were analyzed using two-tailed Student tests. Data are represented as mean ± standard errors of the mean (SEM). The number of independent experiments (*n*) is indicated. A *P* value of less than 0.05 was considered significant. Asterisks throughout indicate *P < 0.05, **P < 0.01, ***P < 0.001 and n.s. a nonsignificant difference.

### Reagents

Unless otherwise specified, all reagents used were Sigma-Aldrich products. IGF1 (PHG0078) was from Thermo-Fisher Scientific (Waltham, MA, USA).

## RESULTS

### Elevated IGFBP7 plasma level observed in naive PWS patients normalizes under GHT, and IGFBP7 decrease negatively correlates with IGF1 increase

**Fig. 1:**
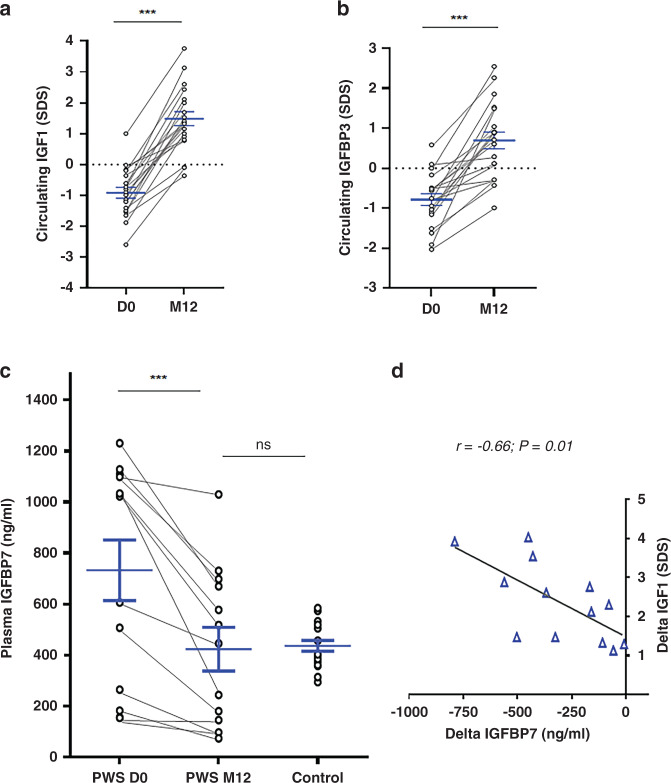
Elevated IGFBP7 plasma levels decrease under growth hormone therapy (GHT) in Prader–Willi syndrome (PWS) patients. (**a**) IGF1 (*n* = 21) and (**b**) IGFBP3 (*n* = 20) values in plasma of PWS patients at day 0 (D0) and after one year (M12) of GHT in 21 PWS patients. (**c**) IGFBP7 values in the plasma of PWS (*n* = 13) at D0 and M12 of GHT, and in age-matched controls (*n* = 18). The immunogen of antibodies used for enzyme-linked immunosorbent assay (ELISA) corresponded to the IGFBP7 sequence (ser28-thr264). Data are represented as mean ± SEM. (**d**) Pearson correlation between IGFBP7 (ng/ml) decreased and IGF1 (SDS) increased within one year of GHT (M12-D0 values) (*n* = 13). Pearson product-moment correlation coefficients (*r*) were used to assess the relationship between variables for PWS patients. ***P < 0.001, ns nonsignificant.

We report here a prospective study in a cohort of 21 young patients with PWS who were naive to GHT (Table 1). We first confirmed the high GH sensitivity of PWS patients, with IGF1 levels increasing fourfold after one year of treatment as ng/ml (50.3 ng/ml, ±34.4 vs. 184.5 ng/ml ± 93.8, *P* < 0.0001) and SDS (−0.92 ± 0.8 *vs*. 1.5 ± 1.04, *P* < 0.0001) (Table 1 and Fig. 1a). As expected, IGFBP3 levels also increased (Fig. 1b). For practical reasons (availability of DXA for very young children), only 11 PWS patients could be evaluated by DXA at baseline and 12 months. Nonetheless, characteristics of these patients were similar to those of the whole cohort, namely regarding their age (Table 1). GHT reduced the fat mass after one year of GHT in patients who were evaluated by DXA (32.3% ± 5.7 vs. 15.2% ± 9.1, *P* < 0.01), with a concomitant positive effect on lean mass (66.6% ± 7.9 vs. 81.8% ± 9.4, *P* < 0.01) (Table 1).

**Table 1:** Clinical and biological characteristics of Prader–Willi syndrome (PWS) patients before and after one year of growth hormone therapy (GHT).

	Baseline	After one year GHT	*p* value
Age (years)	2.1 (0.86–7.79) *n* = 21	3.1 (1.91–8.82) *n* = 21	–
Height (m)	0.79 (0.68–1.1) *n* = 21	0.92 (0.77 –1.17) *n* = 21	*p* < 0.0001
Height (SDS)	−1.24 (−2.66–0.57) *n* = 21	−0.26 (−2.37–1.92) *n* = 21	*p* < 0.0001
BMI *Z*-score	−0.84 (−3.34–1.19) *n* = 21	−0.34 (−3.12–2.19) *n* = 21	0.13
IGF1 (ng/ml)	50.3 (7.2–137.6) *n* = 21	184.5 (25.5–407.7) *n* = 21	*p* < 0.0001
IGF1 (SDS)	−0.92 (−2.58–1) *n* = 21	1.5 (−0.36–3.76) *n* = 21	*p* < 0.0001
IGBFP3 (ng/ml)	2290 (1,237–3,565) *n* = 21	3632.9 (1,874–5,254) *n* = 21	*p* < 0.0001
IGFBP3 (SDS)	−0.8 (−2.03–0.58) *n* = 20	0.6 (−1.28–2.54) *n* = 21	*p* < 0.0001
Patients with DXA analysis			
Age (years)	2.27 (1.04–7.79) *n* = 11	3.35 (2.07–8.82) *n* = 11	–
BMI *Z*-score	−0.8 (−2.24–1.92) *n* = 11	−0.4 (−3.12–2.19) *n* = 11	0.2
Fat mass (%)	32.3 (24.65–46.9) *n* = 11	15.2 (10.4–42.6) *n* = 11	*p* < 0.01
Lean mass (%)	66.6 (44.1–73.68) *n* = 11	81.8 (54.7–89.6) *n* = 11	*p* < 0.01

Twenty-one patients were evaluated. The gender ratio (M/F) was 38%; the genetic diagnosis was as follows: 38% of the patients had deletions, 57% uniparental disomy, and 4% an imprinting defect. Results are presented as mean values (minimum–maximum); IGF1 (SDS) and IGFBP3 (SDS) were calculated according to Feigerlova et al.^13^ IGF1 and IGFBP3 are in ng/ml. Conversion factor for IGF1 from ng/ml to nmol/l is 0.131. Analysis of the body composition by dual-energy X-ray absorptiometry (DXA) was performed in 11 patients.

We investigated IGFBP7, as a potential modulator of the response to IGF1 as stated in the introduction.^14–17^ From the initial cohort, with plasma available, it was possible to test 13 patients for IGFBP7 levels at both baseline and one year of GHT (Table S1). Their characteristics were similar to the cohort of the 21 patients regarding age, sex ratio, and the deletion to disomy ratio. Prior to GHT, IGFBP7 levels were 1.9-fold higher in PWS patients in comparison to healthy subjects of a similar age (727 ng/ml ± 400 vs. 437 ng/ml ± 414, *P* = 0.0003) (Fig. 1c). IGFBP7 levels decreased in response to GHT, declining to the level of controls after one year of treatment (Fig. 1c). Interestingly, the decrease of IGFBP7 level correlated with the increase of IGF1 level in GH-treated PWS patients (*r* = −0.66, *P* = 0.01) (Fig. 1d). Thus, the reciprocal changes in IGF1 and IGFBP7 levels may both contribute to the known high GHT sensitivity of PWS patients and to the effects of GHT on growth and body composition changes.

### *IGFBP7* expression is elevated in iPSC-derived neurons from a PWS patient and a *SNORD116*-deleted patient and decreases under IGF1 stimulation

**Fig. 2:**
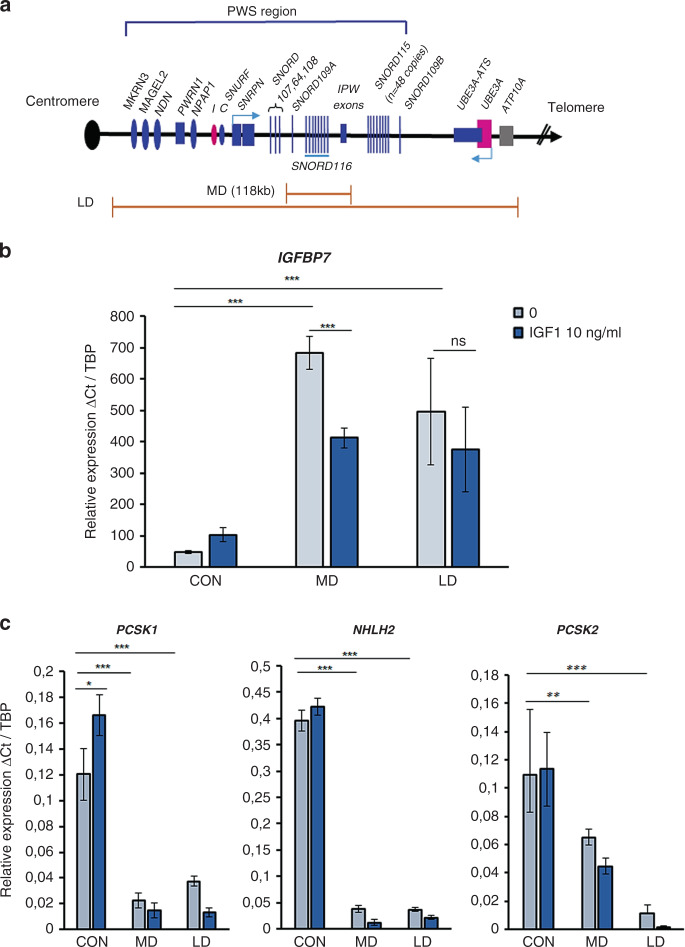
Structure of the Prader–Willi syndrome (PWS) locus and consequence of large deletion (LD) or microdeletion (MD) on *IGFBP7* and proconvertases 1 and 2 (*PCSK1/PCSK2*) and *NHLH2* expression in iPSC-derived neurons at day 34, and responses to IGF1. (**a**) Diagram of the human PWS locus 15q11-q13. Maternally expressed genes are indicated in pink, paternally expressed genes in blue. Protein-coding genes are shown as ovals; small nucleolar RNAs (snoRNAs) as bars (*SNORD116*: 29 copies, *SNORD115*: 48 copies) the imprinting center (IC) is shown by ovals. LD large deletion, MD microdeletion including *SNORD116*. Drawing is not to scale. (**b**) Values of quantitative reverse transcription polymerase chain reaction (qRT-PCR) analysis of *IGFBP7.* (**c**) *PCSK1*, *NHLH2*, and *PCSK2* messenger RNA (mRNA) expression (left, middle, and right, respectively) in untreated induced pluripotent stem cell (iPSC)-derived neurons and treated with IGF1 in phosphate buffered saline (PBS) 10 ng/ml for 2 hours. Data are from a male control subject (CON) (056LB), MD (2 clones: MD-A and MD-C) female, and LD (031MP) female. *TBP* (TATA-binding protein) was used as a housekeeping gene as reference. Results were from three independent experiments. The comparison was assessed by a two-tailed Student’s *t-*test. Data are expressed as mean ± SEM. *P < 0.05, **P < 0.01, ***P < 0.001. ns nonsignificant.

Given the potential interest of iPSC-derived human neurons in PWS pathophysiology as described previously,^8^ we tested the hypothesis that *IGFBP7* expression was high in these cells. Characteristics of patients with a large deletion (LD) or with a microdeletion including *SNORD116* (MD)^5^ and from whom the skin fibroblasts were obtained are described in Table S2 and Fig. 2a. IPSC-derived neurons retained the molecular signatures of PWS, including the absence of *SNORD116* expression.^27^ The induction of neuronal differentiation from iPSCs was validated by testing several gene expressed in mature neurons, including *NR4A2*, *DRD2*, *GIRK2*, and *TH* (Fig. S1). CD56 + iPSC-derived neurons were selected by flow cytometry. We observed an increase in *IGFBP7* mRNA expression in iPSC-derived neurons from MD and LD patients compared to controls (683 ± 52, 496 ± 169 respectively vs. 47.6 ± 4.75; *P* < 0.0001) (Fig. 2b). It is important to note that this was the case in neurons from the *SNORD116-*deleted MD patient.

Considering the relationship between decreased IGFBP7 and increased IGF1 plasma concentrations in GH-treated patients (Fig. 1c), we tested the effect of IGF1 on iPSC-derived neurons. IGF1 treatment (10 ng/ml) did not change the expression of the MCR genes (*SNORD116*, *IPW*, and *SNORD109A*) in iPSC-derived neurons from healthy people (Fig. S2). Notably, *IGFBP7* expression decreased by 40% under IGF1 in MD neurons (*p* < 0.001). In LD neurons the decrease was of 25%, which was not significant (Fig. 2b).

We also tested the genes involved in prohormone processing,^8^ as they can potentially impact the production of the intact form of IGFBP7.^18–21^ In agreement with previous data, *PCSK1* and *NHLH2* expressions were lower in MD and LD iPSC-derived neurons than in control neurons (Fig. 2c). Moreover, *PCSK2* expression was also lower (Fig. 2c). IGF1 slightly increased *PCSK1* expression in the control iPSC-derived neurons but not in those from LD and MD patients and did not affect *PCSK2* and *NHLH2* expression in either LD and MD neurons or control cells (Fig. 2c). Interestingly, *PCSK1*^8^ and *PCSK*2 could have an effect on IGFBP7 cleavage that influences its activity.^18–21^

### IGFBP7 expression is not modified by an editing mechanism in PWS patients

**Fig. 3:**
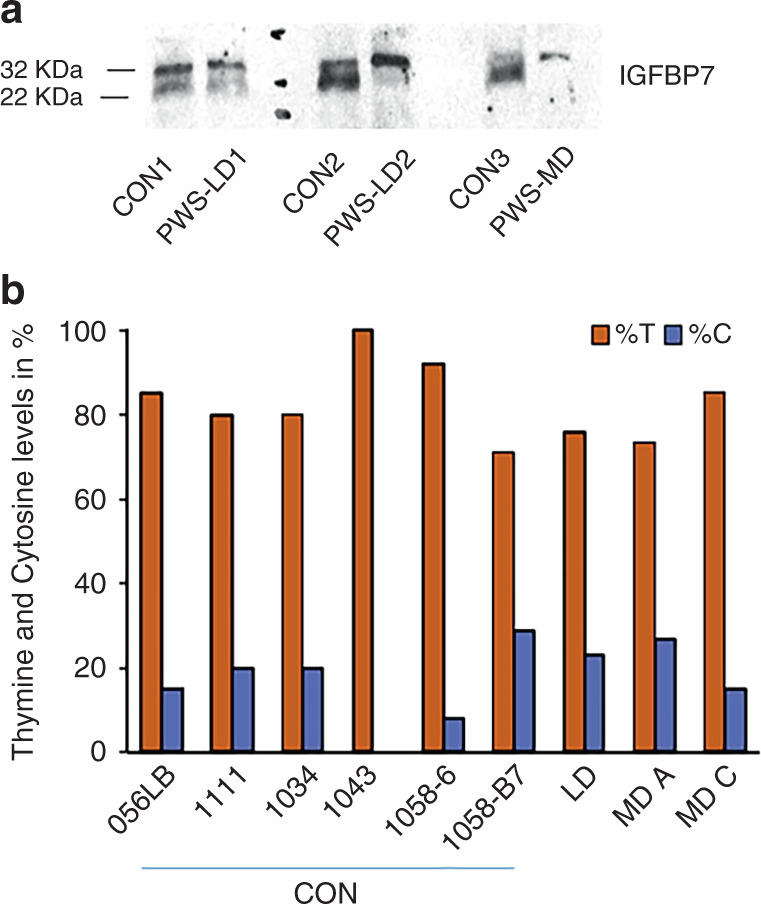
Intact IGFBP7 expression is increased in large deletion (LD) and microdeletion (MD) cells, but there is no difference in IGFBP7 editing between controls and Prader–Willi syndrome (PWS) patients in induced pluripotent stem cell (iPSC)-derived neurons. (**a**) Western blot of IGFBP7 secreted in a medium of immortalized fibroblasts from controls and PWS subjects cultured for 96 hours. 50 µg of total protein were deposited per well. Detection was performed with antibodies directed against the (Ser28-Thr264) IGFBP7 sequence, which detect both intact (32 kDa) and cleaved (22 KDa) forms. The cleaved form was less detected in LD and MD cell extracts than in controls. PWS-LD large deletion, PWS-MD PWS microdeletion. (**b**) Nucleotide frequencies from RNA sequencing of raw data from Burnett et al.^8^ performed at the position hg19: chr4:57,976,229 (hg38: chr4:57,110,063), which includes the A to I editing site of IGFBP7 at codon 95, hypothetical target for proteases and proconvertase 1. Comparison between iPSC-derived neurons from 6 unaffected subjects (CON) (056LB, 1111, 1034, 1043, 1058-6, and 1058-B7), 1 PWS patient with large deletion LD (031MP), and 1 PWS patient with microdeletion (MD) (2 clones were used: MD A and MD C). Readings were mapped to a reference genome (human: NCBI/build37.2).

As described for the *SNORD115* for modulation of *5-HT2c* receptor editing,^31^ *SNORD116* deletion could impact *IGFBP7* editing with consequences on its cleavage by proteases.^18–21^ Notably, PC1, product of *PCSK1*, could theoretically cleave at a furin site (R/K cleavage site at positions 94/95, Fig. S3).^32^ Thus, we first analyzed the presence of uncleaved and cleaved forms of IGFBP7 in the secretion medium of fibroblasts of PWS patients and controls. The uncleaved form of IGFBP7 was found to be more abundant in LD and MD cells (Fig. 3a). We analyzed by RNA sequencing the editing of the sequence potentially involved in IGFBP7 cleavage by PC1 (Fig. 3b). The nucleotide frequencies were comparable in LD, MD, and control neurons. A similar editing profile among control and PWS cells was observed in fibroblasts and adipocytes (Fig. S4). Thus, the editing process does not appear to be involved in the increased abundance of intact IGFBP7 in LD and MD cells.

### *Igfbp7* expression in a mouse model of *Snord116*-deleted *PWScr*^*p−/m+*^ mice mimics *IGFBP7* expression in the PWS patient with LD and in the *SNORD116*-deleted MD patient

**Fig. 4:**
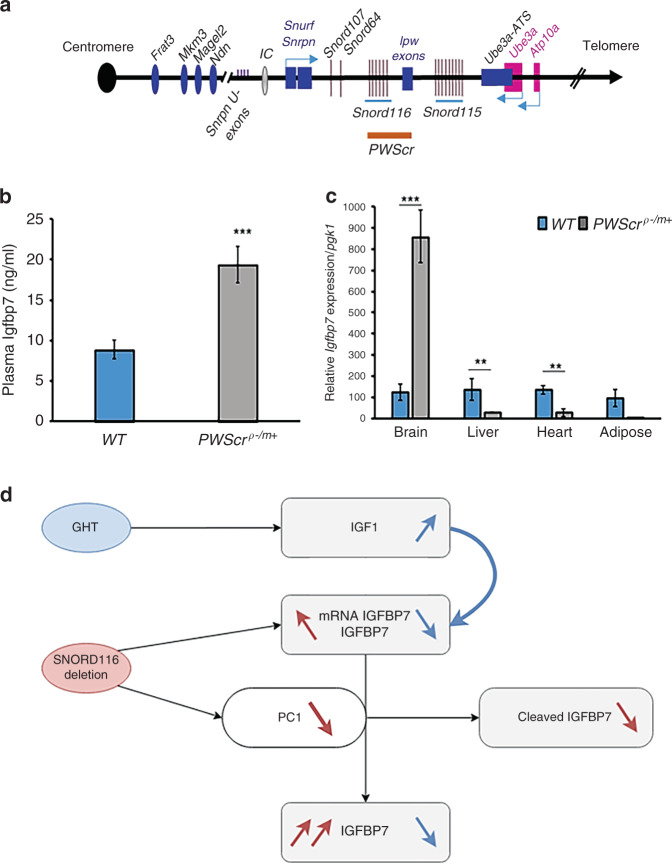
IGFBP7 is elevated in the plasma and brain of *PWScr*^*p−/m+*^ mice and proposed schema of IGFBP7 regulation by *SNORD116* deletion. (**a**) Schematic representation of the orthologous PWS region on mouse chromosome 7qC. Small nucleolar RNAs (snoRNA) genes are represented by bars (*Snord116*: 71 copies and *Snord115*: 136 copies), other genes locations are depicted with rectangles and ovals. Paternally expressed genes are indicated in blue, maternally expressed genes in pink. The imprinting center (IC) is shown by a gray oval. The orange line indicates the deleted area in the studied model of *PWScr*^*p−/m+*^ mice. Drawing is not to scale. (**b**) Circulating Igfbp7 was 2.2-fold higher (*P* = 0.00034) in P7 *PWScr*^*p−/m+*^ mice (*n* = 7) compared to wild-type (WT) littermates (*n* = 7). Normality was assessed by Shapiro–Wilk normality tests, and statistical differences were analyzed using the paired Student’s *t-*test. Data are expressed as mean ± SEM, ***P < 0.001. (**c**) Transcript levels of *Igfbp7* in different mouse tissues *PWScr*^*p−/m+*^ (*n* = 7) compared to WT littermates (*n* = 7). *Igfbp7* were highly expressed in *PWScr*^*p−/m+*^ brains. Data are shown as mean delta Ct values compared to *pgk1* as the reference gene giving the more stable expression besides other housekeeping genes. A comparison was drawn by a 2-tailed Student’s *t-*test. Data are expressed as mean ± SEM. **P < 0.01, ***P < 0.001. (**d**) Proposed schema of IGFBP7 regulation by *SNORD116* deletion and GHT in PWS. Both increased expression of *IGFBP7* and proconvertase *PCSK1*/ PC1 deficiency could explain the raised IGFBP7 level in PWS patients, IGFBP7 being a potential target of PC1, which is downregulated in *SNORD116*-deleted neurons. Loss of *SNORD116* results in an increase in *IGFBP7* mRNA expression and a decrease in potential IGFBP7 cleavage by PC1, with high levels of secreted IGFBP7 in naive PWS patients. Growth hormone therapy (GHT) stimulates IGF1 production, which downregulates *IGFBP7* mRNA expression and lowers its circulating level.

As stated above, the MD patient (deletion of the *SNORD116* cluster) displays many similarities to other PWS patients, including hyperphagia and obesity in childhood.^5^ Mice that lack the paternal PWS critical region (*PWScr*^*p−/m*+^), which harbors the *Snord116* cluster in the 7qC region homologous to the human 15q11-13, have been created by Skryabin et al.^30^ (Fig. 4a). The *PWScr*^*p−/m+*^ phenotype includes growth retardation and low Igf1, mild hyperphagia and impaired satiation.^33^ These results led us to analyze Igfbp7 in *PWScr*^*p−/m+*^ mice. Similarly to findings observed in PWS patients, Igfbp7 plasma levels were higher in *PWScr*^*p−/m+*^ mice than in WT mice, with a 2.2-fold increase (19.3 ng/ml ± 8.5 vs. 8.81 ng/ml ± 4.2, *P* = 0.0003) (Fig. 4b). Interestingly, *Igfbp7* mRNA expression in *PWScr*^*p−/m+*^ mice was increased only in the brain, and not in the liver, heart, and adipose tissue, these tissues displayed even lower levels compared to WT mice (Fig. 4c). Therefore, increased *Igfbp7* expression appears to be a characteristic of brain tissue in *PWScr*^*p−/m+*^ mice.

## DISCUSSION

Early GHT is a major option for young patients with PWS,^11^ its main consequence being elevated IGF1 levels.^13^ In this study, IGF1 plasma levels increased fourfold under GHT in a prospective study of young PWS patients naive to GHT. Notably, besides its effect on growth, GHT also resulted in improvement of BMI, decreased fat mass, and increased lean mass, which confirmed the positive effect of GHT.

As stated in the Introduction, we further explored IGFBP7 as a potential modulator of IGF1R activation.^14–17^ IGFBP7 levels were higher in naive PWS patients versus control population, and levels normalized with GHT. The IGFBP7 decrease correlated with IGF1 increase after one year of GHT. This suggests that high GHT sensitivity might be associated with a lowering of IGFBP7 levels since, in most cases, IGFBP7 hinders IGF1R activation.^17^ Indeed, the levels of IGFBP7 in naive patients were not homogeneous. However, it can be noted that among four PWS patients who had the initial lowest IGFBP7 values, in three of them IGFBP7 decreased under GHT. Indeed, multiple factors may be implicated in phenotypical heterogeneity of PWS patients. Of note, we found no difference between disomy and deletion patterns (730 vs. 735 ng/ml respectively). As to the control group, it was homogeneous like in other studies.^34,35^ However, values observed in adult patients usually are lower than those in our control subjects^34,35^ suggesting possible fluctuations of IGFBP7 levels with age. Plasma IGFBP7 values were also high in the model of *PWScr*^*p−/m+*^ mice deleted for *Snord116*. Values were different from humans, which can be due to physiological particularities, or to characteristics of the assays used. Clearly, this preliminary data will require investigation in larger PWS cohorts.

We also tested *IGFBP7* expression in iPSC-derived hypothalamic cells and in the *PWScr*^*p−/m+*^ mice. It was increased in PWS hypothalamic neurons, including in *SNORD116-*deleted MD cells, and in the brains from *PWScr*^*p−/m+*^ mice. This suggest the implication of *SNORD116* in *IGFBP7* expression in hypothalamic cells. To our knowledge, data regarding the comparative expression of IGFBP7 between humans (so far described as relatively ubiquitous^36^) and mice, are scarce, but it can be considered similar in many organs. The comparative expression in hypothalamic cells is not well documented in available data sets. Nevertheless, we found IGFBP7 to be elevated in brain but not in the liver, heart, or adipose tissue from *PWScr*^*p−/m+*^ mice. Therefore, comparison is uneasy but our data are supportive of altered expression patterns of *IGFBP7* both in mice and humans PWS neurons.

At protein level, intact and cleaved IGFBP7 display different biological activities.^14,18–21^ We found a lower expression of proconvertases *PCSK1*/PC1, *PCSK2*/PC2, in PWS neurons, which could influence IGFBP7 cleavage. PWS cells, including *SNORD116*-deleted MD cells, displayed high abundance of intact IGFBP7 and lower expression of the cleaved form. Since *IGFBP7* editing could suppress a cleavage site for proteases^18–21^ (namely for PC1 and PC2), we analyzed the nucleotide frequencies but observed no difference between LD or MD cells and controls, namely in iPSC-derived neuronal cultures. Therefore, the lower expression of *PCK1* and *PCSK2* could partly explain the increase of the intact form of IGFBP7 in PWS cells but without the implication of mRNA editing. Notably, we did not find the same molecular apparent weight of the cleaved form (22 KDa) as in Ahmed et al. (25 KDa) and Chen et al. (27 KDa) but they were close. Indeed, as we have not sequenced the cleavage site in our cells, we cannot infer that PC1/PC2 proteases are active on IGFBP7. Moreover, we did not analyze the abundance of PC1 and PC2 at the protein level, therefore other proteases like matriptases could be involved.^17^ In addition, IGFBP7 is not considered to be a prohormone, thereby, the downregulation of *PCSK1* and *PCSK2* in PWS cells, especially in *SNORD116*-deleted cells, only remains a hypothesis to explain the increase in intact IGFBP7.

Clearly, the inverse relationship between IGF1 and IGFBP7 levels is highly intriguing, although its mechanism is presently unknown, which suggests more investigations in the future. It would therefore be interesting to test the level of activation of intermediate pathways such as IGF1R/ MAP Kinase and PI3 Kinase under IGF1. On the other hand, *SNORD116* may directly impact transcription, through an epigenetic mechanism,^7^ which is important for regulation of *IGFBP7* expression by methylation.^24^ As stated above, IGFBP7, interacting with IGF1R, displays an ambiguous action with regard to cell proliferation.^14^ Since IGFBP7 is known to block the effect of IGF1R,^17^ it seems logical that the increase in IGF1 leads to a decrease in IGFBP7 abundance under GHT, as cells would not concomitantly activate the accelerator and the brake of cell proliferation.

Therefore, two independent mechanisms may lead to increased secretion of intact IGFBP7 in *SNORD116*-deleted and PWS cells. A lack of *SNORD116* expression might result in increased *IGFPB7* expression as observed here in hypothalamic cells and in the brains of *PWScr*^*p−/m+*^ mice. Secondly, the decrease of *PCSK1*/PC1 expression responsible for decreased IGFBP7 proteolysis could also result in an increased expression of the IGFBP7 intact active form. Figure 4d summarizes these results.

A major result is that increased *IGFBP7* expression is specifically observed in PWS neuronal cells and decreased under IGF1. High IGFBP7 levels may be involved in degenerative mechanisms^25,26^ and, indeed, PWS is now considered as a degenerative disease.^10^ A potential positive effect of IGFBP7 modulation in the progression of neurodegeneration has been suggested.^25^ Alternatively, IGFBP7 levels have been associated with a decrease in infant brain volume.^37^ Therefore, decreasing IGFBP7 levels under GHT may have positive effects on the cognition of PWS patients.^38^

Finally, IGFBP7 is described as a proapoptotic factor that is downregulated in several cancer lines.^14,24^ Theoretically, lowering the IGFBP7 level could raise the question of increased cancer risk. A survey including 1,077 patients did not show any increase in the cancer rate in PWS patients, except for acute myeloid leukemia (AML).^39^ It is one case where IGFBP7 could act as an oncogene,^14^ AML being associated with high IGFBP7 levels in bone marrow as a marker of severity.^40^ Therefore, the higher risk of AML in PWS patients could be due to high levels of IGFBP7, with GHT conferring protection by reducing circulating IGFBP7 levels.

In this study, IGFBP7 levels were elevated in the plasma of PWS patients and *PWScr*^*p−/m+*^ mice. *IGFBP7* was also overexpressed in iPSC-derived *SNORD116*-deleted neurons, and in the brain of *PWScr*^*p−/m+*^ mice. IGFBP7 levels decreased with GHT in vivo and with  IGF1 in vitro, which suggests a beneficial effect of GHT on the IGFBP7 status of PWS patients. Overall, our data suggest that *SNORD116* and GHT could impact IGFBP7 levels in PWS, which will necessitate further investigations.

## Supplementary information

Supplementary Information

Supplementary Table

## Data Availability

The data that support the findings of this study will be available upon request.
