# Supplementary material for: SNORD116 and growth hormone therapy impact IGFBP7 in Prader–Willi syndrome
Source: Genet Med. 2021 May 26;23(9):1664–72. doi: 10.1038/s41436-021-01185-y (PMC8460435; doi:10.1038/s41436-021-01185-y)
Supplement: Supplementary file 1 — Supplementary Information [file 41436_2021_1185_MOESM1_ESM.docx]

SUPPLEMENTARY INFORMATION

**Supplementary Materials and Methods**

**Criteria for the NCT01298180 clinical trial:** Participants were outpatients and inpatients from the French Reference Center for PWS in Toulouse, as well as 16 other sites in France.

The inclusion criteria of PWS patients were as follows: female or male child of age > or = 1 year; child naive for GH treatment and who must begin treatment with GH; child covered by a national insurance scheme or an equivalent; signature of the informed consent by one of the holders of parental authority.

The inclusion criteria of controls were as follows: female or male child of age > or = 1 year; child paired for age (+/-1 year) and sex with regard to the PWS group. Child hospitalized at the Children’s hospital of the University Hospital of Toulouse for a programmed surgical operation, child covered by a national insurance scheme or an equivalent; signature of the informed consent by one of the holders of parental authority.

The exclusion criteria of PWS patients were as follows: Child presenting a contraindication to growth hormone; welded growth cartilage; tumoral pathology in process of evolution; corticosteroid therapy (not substitute); known allergy to the solvent; poorly controlled diabetes, child presenting hypersensitivity to the active principle or one of the excipients of Genotonorm ® or Omnitrope ®; child presenting severe obesity (defined by a reported weight/size> 200 %); child presenting clinical ENT signs (snores associated with hypertrophy of the adenoids and\or the tonsils); child presenting clinical signs evoking a respiratory sleep disorder (night-respiratory snores, respiratory pauses during sleep).

The exclusion criteria of controls included: chronic pathology in which an abnormality of growth would be involved; other hormonal abnormalities; children receiving long-term treatment, particularly corticosteroid therapy, likely to interfere with the sensitivity to GH or insulin; holder of the parental authority under supervision, guardianship or under judicial protection; simultaneous participation in another study.

**DXA analysis**: DXA works on the principle that body tissues (fat, muscle, bone) attenuate X-ray differently. Lunar-intelligent DXA (iDXA) like other DXA device measure attenuation of X-rays at both low and high energy synchronously. Each pixel is attributed an *R-*Value, each tissue possessing a specific signature. The pixel density of Lunar iDXA images results from 100 kV energy, which provides good performance, resolution and precisions with CV<1% (Hind *et al.* Eur J Clin Nutr 2010). Lunar iDXA software (Infant whole body analysis enCore 2007 software) provides absolute and relative values for body composition variables. Lunar iDXA has been evaluated for analysis of body composition in infants (Fields *et al.* Obesity 2012). We expressed fat and lean mass of total body as percent of the total body mass. See supplementary references below.

e infant was placed supine on the scanning bed

wearing only a disposable diaper while swaddled in a light cotton blanket

provided by the laboratory. Typically, the infant remained awake during

the procedure although the lights were o as an animated movie played

on a portable DVD player outside of the scanning eld. No infant was

re-scanned. Body composition variables were calculated using standard

procedures outlined in the procedure manual.

**Fibroblast reprogramming to iPSCs:** Primary human fibroblasts from unaffected controls and PWS patients were reprogrammed to iPSCs using retrovirus reprogramming, Sendai virus reprogramming, or mRNA reprogramming. They were provided by R Leibel’s team and performed as described in Burnett et al. 2016 ^27^.

**Differentiation of iPSCs into neurons**: The method for the induction of the differentiation of iPSCs into neurons was described previously^27^. In brief, iPSC cultures were routinely retained on MEFs (Globalstem) and cultured in human ES media: KO-DMEM (Thermo Fisher Scientific), 10% KO-SR (Thermo Fisher Scientific), 1% NEAA, 1% glutamax, 0.1% β-ME, 1% P/S, and 10 ng/mL bFGF. Media was supplemented with Y-27632 upon passaging. The medium was changed daily. iPSCs were grown until they reached the subconfluent state. IPSCs were differentiated into neurons using a modified SMAD inhibition protocol from fibroblasts from PWS patients and control patients^26^. These undifferentiated cells cultured on MEFs (mouse embryonic fibroblasts) were dissociated and subcultured onto fresh MEFs at a density of 1,000,000 cells in a petri dish in HES medium with Y-27632 on day 0. On day 1: the medium was changed to EB medium (HES medium without bFGF), to which we added the LSB, consisting of 10 μM of SB431542 (Stemgent) and 250 nM of LDN193189 (Stemgent). On day 2 and day 3, the cells were treated with EB + LSB medium, and 70% of the volume was changed every day. On day 4: the medium was composed of 75% EB medium + 25% N2 medium (Thermo Fisher Scientific) + LSB. On day 5: 50% EB medium + 50% N2 medium + LSB. On day 6: 25% EB medium + 75% N2 medium with LSB. From day 7 to day 10: N2 medium with LSB. On day 10, the cells were dissociated and subcultured on plates covered with poly-L-ornithine (Sigma-Aldrich) and laminin (Sigma-Aldrich), then placed in an N2 medium with LSB + rock inhibitor. 200,000 cells per well for a 6-well plate. On day 11 the medium was changed to N2 to which were added B27 (Thermo Fisher Scientific) without retinoic acid and 20ng /μL of recombinant BDNF (Miltenyi). Neurons were harvested on day 34 for RNA extraction. *NR4A2, DRD2, GIRK2,* and *TH* genes were tested in order to validate the cells' differentiation. Primers used are detailed in **Table S3** in Supplementary data.

**Isolation of iPSC-derived neurons:** Cells were dissociated with Dispase II (Sigma) and filtered through a 35-mm cell strainer (BD Biosciences) to obtain a single-cell suspension. Cells were then suspended in 100μl of a sterile iPSCs staining buffer containing D-PBS containing 0.5% BSA fraction V (Invitrogen), 1% penicillin/streptomycin (Invitrogen), 2 mM EDTA (Invitrogen), and 20 mM glucose (Sigma-Aldrich). Cells were stained live for 20 min with a CD56-V450 (1 μl; BD Biosciences, 560360) or Stem Cell Technologies Anti-Human CD56 (NCAM) antibody (60021AZ) was added to the cells and incubated at room temperature for 15 minutes protected from light. The stained cells were washed once with iPSCs staining buffer and sorted immediately on a 5 laser BD Biosciences Cell Sorter. Cells were sorted into a 15 ml tube containing growth media.

**Western blot analyses:** After 96 hours of seeding fibroblasts from PWS patients and CON subjects*, s*ecreted media were lysed in a buffer containing 25 mM Tris, pH 7.4, 50 mM KCl, 0.5 mM EDTA, 5 % glycerol, 0.5% Triton X-100, 20 mM NaF, 2mM Na_3_VO_4_, and protease inhibitors. The lysates were cleared by centrifugation and total proteins were measured using the Bradford method (Bio-Rad). Lysate aliquots containing 50 µg of proteins were diluted in a Laemmli sample buffer, boiled, and processed for immunoblotting according to a standard procedure. The IGFBP7 antibody from Cell Signaling (#97884) was directed against C-terminal IGFBP7, detecting total IGFBP7. Monoclonal anti-rabbit horseradish peroxidase-conjugated antibodies were from Sigma Aldrich Co. Horseradish peroxidase (HRP)-conjugated secondary antibodies were from Sigma Aldrich Co. Blots were revealed by an enhanced chemiluminescence detection system (Amersham). All PWS cases and Controls (CON) were analyzed in the same experiment.

**ddPCR reactions:** All steps of ddPCR were carried out on the BioRad QX200 system. PCR reactions were set up as follows: 11 μl of Probe Mastermix, 1.1 μl of fluorescent probes, 2 μl of template, and the remainder made up to 22 μl with PCR-grade water. Droplets were formed in sets of 8 using the BioRad Droplet maker with 70μl of droplet-generating probe oil. Droplets were transferred to a BioRad 96 well PCR plate, and the PCR reaction was as follows: 95°C for 10 minutes, 94°C for 30 seconds, 59°C for 1 minute, 40 cycles of steps 2-4, and 98°C for 10 minutes. Droplets were then read on the BioRad droplet reader. Thresholds were set between positive and negative droplets based on template controls. BioRad QuantaSoft software reports concentration in copy per μl of reaction mixture. We used this value to calculate ratios between the 2 alleles. Technical replication may not always be necessary for ddPCR, considering that the Poisson Error calculated by the BioRad software can be used as an estimate of experimental error (BioRad ddPCR Applications Guide).

Supplementary figures:

Supplementary Table S1: Clinical and biological characteristics of the PWS patients and control subjects.

(1: female; 2: male; MA: Methylation abnormality; Age expressed in years; FM: Fat Mass; LM: Lean Mass). IGF1, IGFBP3 and IGFBP7 [at Day 0 (_0) and after twelve months (_12)] are in ng/ml. Conversion factor for IGF1 ng/ml to nmol/l is 0.131. Data are provided as an Excel Table.

**Supplementary Table S2. Anthropometric data of patients and an unaffected control from which iPSCs and iPSC-derived neurons were generated.**

^a^ Patient was still in 'the failure to thrive' phase at the time of biopsy; maximum lifetime BMI not available.

| **Cell Line** | **Genotype** | **Name** | **Sex** | **BMI at biopsy or maximum lifetime** |
| --- | --- | --- | --- | --- |
| 056LB | Unaffected Control | CON | M | 19.6 |
| 031MP | PWS Large Deletion | LD | F | 15.52^a^ |
| 066MD | PWS Microdeletion (2 clones; MD A and MD C) | MD | F | 55.4 |

**Supplementary Table S3. Primers used for qRT-PCR analysis.**

| **Human** | **Forward** | **Reverse** |
| --- | --- | --- |
| ***IGFBP7*** | GCGAGCAAGGTCCTTCCATA | GGGATTCCGATGACCTCACA |
| ***SNORD116*** | CGATGATGAGTCCCCCATAAAAAC | CAGTTCCGATGAGAACGACG |
| ***SNORD109*** | ATAATTGTCTGAGGATGCT | GATTGACATCTGGAATGAGTC |
| ***IPW*** | TGCCTAGACCACCCACTAAAGG | AGTCTCCATGCGGAAGGAAGA |
| ***NHLH2*** | GTCCGGACTCAGCATCATTT | ATATTTTCCGGAATCTCCCCT |
| ***PCSK1*** | ACCAGGTGCTGCATATCTCG | CACAATGACTGCACGGAGAC |
| ***PCSK2*** | TTTCGGTCAAATCCTTCCTG | TGCAAAGGCCAAGAGAAGAC |
| ***TBP*** | GGCTGGCTAGGGATGAAGA | GGCGTCTGGACTAGGAGCTT3 |
| ***NR4A2*** | CAGGCGTTTTCGAGGAAAT | GAGACGCGGAGAACTCCTAA |
| ***DRD2*** | GCAGACCACCACCAACTACC | CCACTCACCTACCACCTCCA |
| ***GIRK2*** | CACATCAGCCGAGATCGGAC | GGTAGCGATAGGTCTCCCTCA |
| ***TH*** | TCATCACCTGGTCACCAAGTT | GGTCGCCGTGCCTGTACT |
|  |  |  |
| **Mouse** | **Forward** | **Reverse** |
| ***Igfbp7*** | AAGAGGCGGAAGGGTAAAGC | TGGGGTAGGTGATGCCGTT |
| ***Pgk1*** | CTGACTTTGGACAAGCTGGACG | GCAGCCTTGATCCTTTGGTTG |

Supplementary Figure S1. Characterization of iPSCs-derived neurons, control (CON), MD, and LD patients. The mRNA expression levels of *NR4A2*, *DRD2*, *GIRK2,* and *TH* were analyzed before (iPS) and after neural induction at D36 (Neu) by real-time PCR (n=3). The quantified results were normalized against TBP mRNA levels.


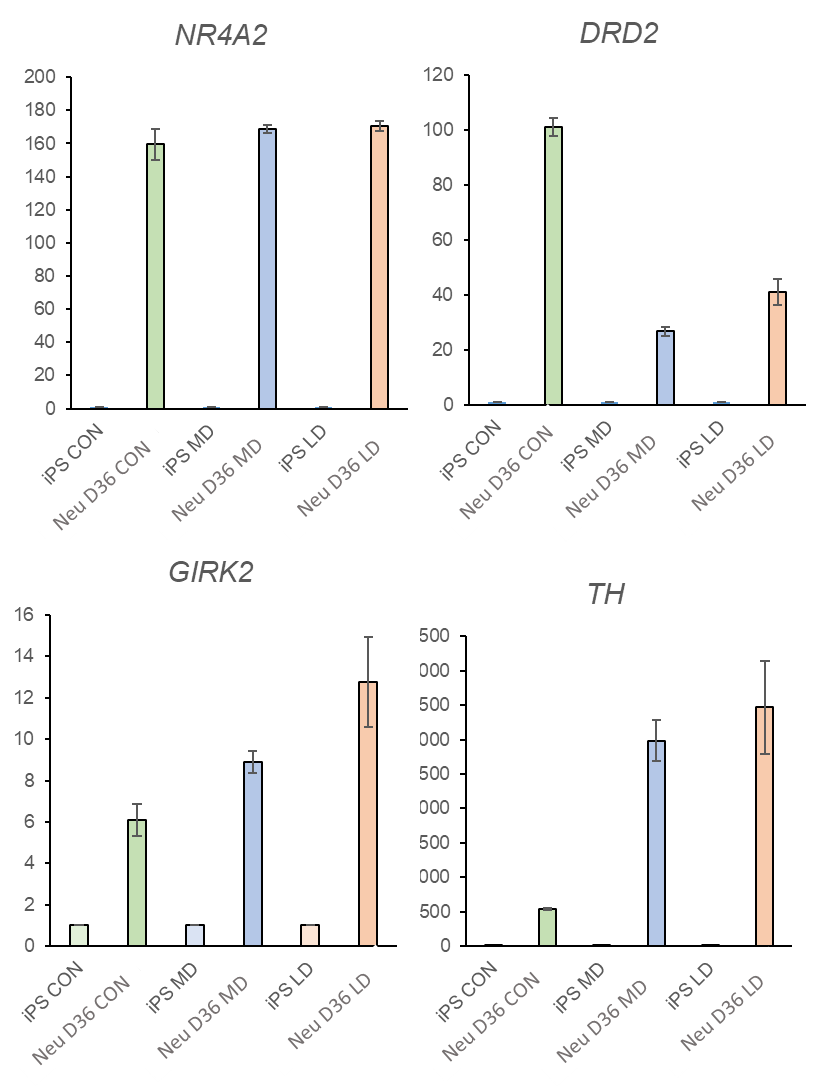


**Supplementary Figure S2. IPSC-derived neurons from large deletion (LD) and microdeletion (MD) PWS patients as expected, display reduced gene copy numbers in the PWS loci and IGF1 has no effect on the copy number of *SNORD116*, *IPW* and *SNORD109A*.** Blue bars represent untreated cell samples and orange bars IGF1-treated samples. *SNORD11*6, *IPW* and *SNORD109A* are deleted in both large-deletion (LD) and microdeletion (MD).


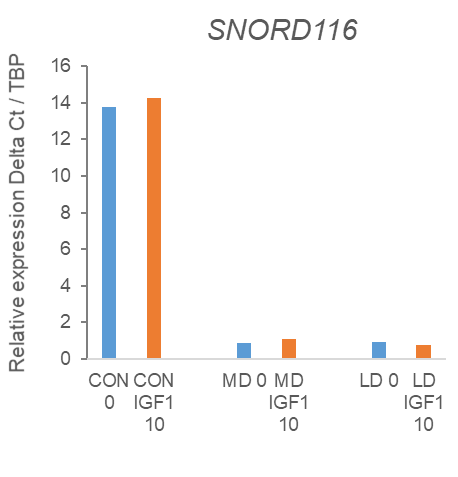

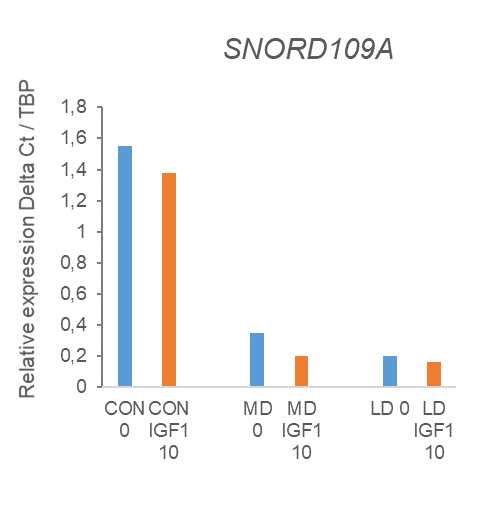

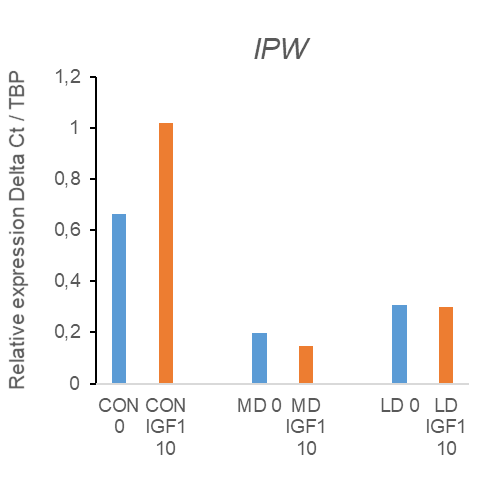


**Supplementary Figure S3. Predicted Furin pro-protease cleavage sites such as PC1 cleavage site using the ProP 1.0 Server at the 94 position: KSRKRR/KG, Arg (R)/ Lys (K).** We used the ProP 1.0 software to examine the existence of potential sites for the cleavage of IGFBP7 by PC1. A potential R/K cleavage site for PC1 (underlined below) exists at positions 94/95 within a VKSRKRR/KGAG sequence in the IGFBP7 sequence ^18–21^. The protease cleavage site at lysine 95 has potential consequences for the functionality of IGFBP7 and its processed peptides^32^. Thus, change of the codon 95 from AAG to AIG by an editing mechanism could theoretically result in K95R substitution, leading to a decreased IGFBP7 proteolysis by PC1 in PWS cells.


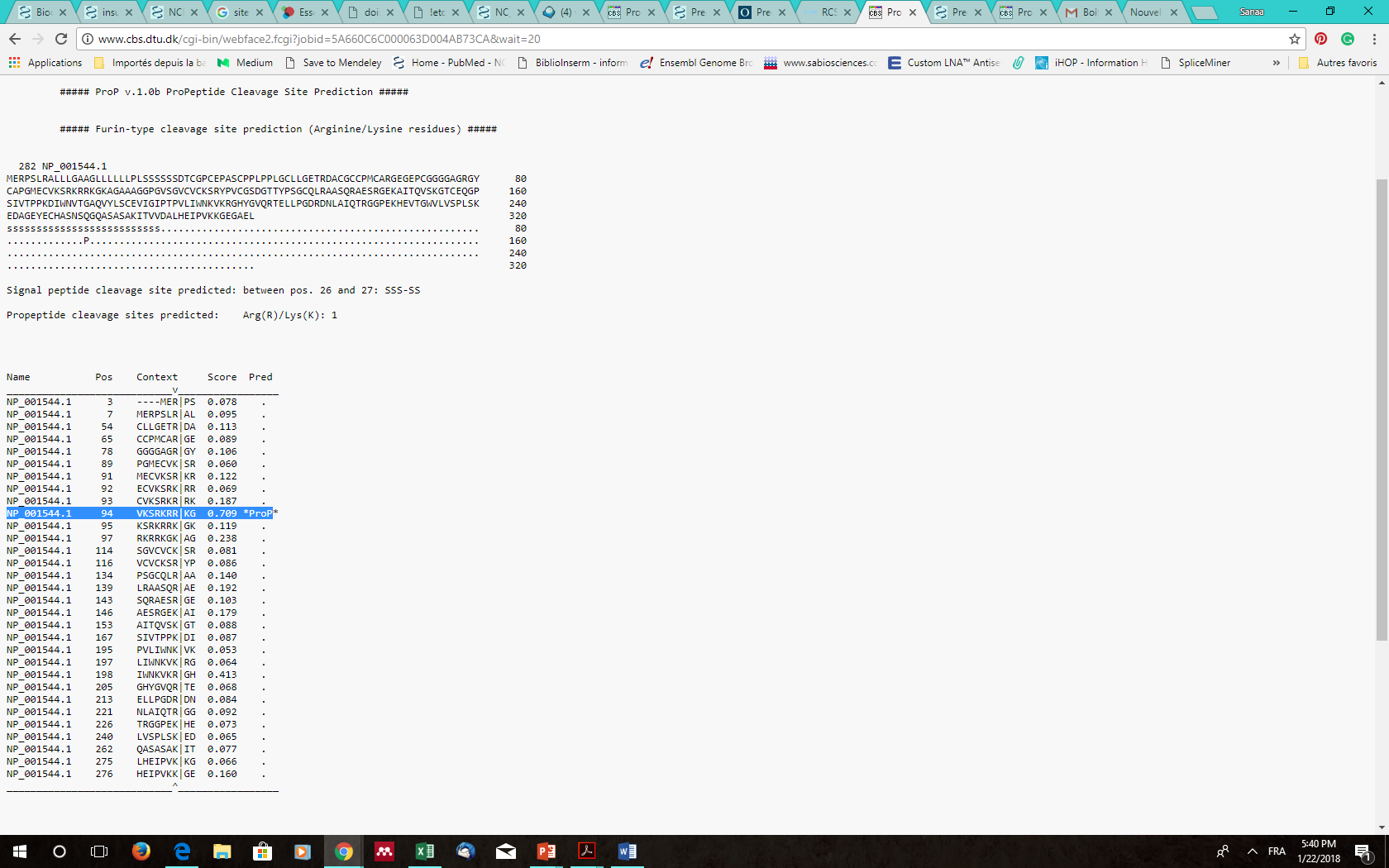


**Supplementary Figure S4. The editing profile of IGFBP7 in adipocytes and fibroblasts is not different from that of PWS patients and controls and not modified by IGF1 treatment.** Copy number validation by droplet digital PCR study of the editing profile of IGFBP7 in adipocytes and fibroblasts from 5 CON and PWS patients :3 PWS-Disomy (Dis) (adipocytes and fibroblasts), 3 PWS-Deletion (Del) (adipocytes and fibroblasts) and 1MD (fibroblasts). The panels show the percentage for the ratio of mutated alleles on wild alleles represented by fractional abundance of the edited site of IGFBP7 as processed by BioRad QuantaSoft^™^. The error bars represent the maximum and minimum Poisson distribution for the 95% confidence interval. The sequence that retains the putative VKSRKRR/KGAG theoretical cleavage site for PC1 was the highest expressed in all cells.


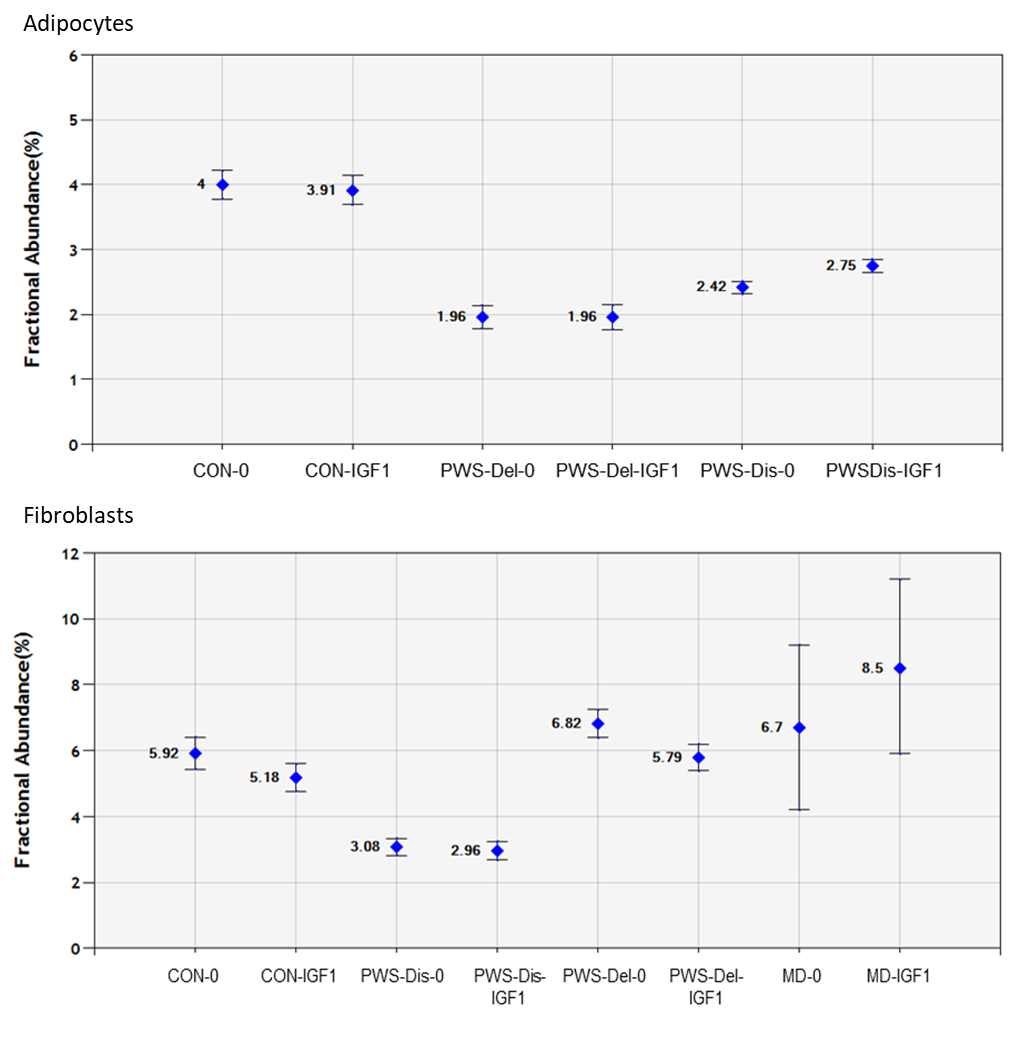


**Supplemental references**

Hind K, Oldroyd B and Truscott JG. *In vivo* precision of the GE Lunar iDXA densitometer for the measurement of total body composition and fat distribution in adults. *Eur J Clin Nutr*. 2011; 65, 140–142.

Fields DA, Demerath EW, Pietrobelli A and Chandler-Laney PC. Body Composition at 6 months of Life: Comparison of air displacement plethysmography and dual-energy X-ray absorptiometry. *Obesity*. 2012; 20, 2302–2306. doi:10.1038/oby.2012.102.
